# Antibacterial Activity, Antioxidant Effect and Chemical Composition of Propolis from the Región del Maule, Central Chile

**DOI:** 10.3390/molecules201018144

**Published:** 2015-10-06

**Authors:** Nélida Nina, Cristina Quispe, Felipe Jiménez-Aspee, Cristina Theoduloz, Gabriela Egly Feresín, Beatriz Lima, Elba Leiva, Guillermo Schmeda-Hirschmann

**Affiliations:** 1 Laboratorio de Química de Productos Naturales, Instituto de Química de Recursos Naturales, Universidad de Talca, Casilla 747, Talca 3460000, Chile; E-Mails: vica17_a@hotmail.com (N.N.); elquispe@unap.cl (C.Q.); fjimenez@utalca.cl (F.J.-A.); 2 Facultad de Ciencias de la Salud, Programa de Magister en Ciencias Biomédicas, Universidad de Talca, Talca 3460000, Chile; E-Mail: eleivam@utalca.cl; 3 Facultad de Ciencias de la Salud, Universidad Arturo Prat, Casilla 121, Iquique 1110939, Chile; 4 Laboratorio de Cultivo Celular, Facultad de Ciencias de la Salud, Universidad de Talca, Casilla 747, Talca 3460000, Chile; E-Mail: ctheodul@utalca.cl; 5 Instituto de Biotecnología, Facultad de Ingeniería, Universidad Nacional de San Juan, Av. Libertador General San Martin 1109 (oeste), San Juan 5400, Argentina; E-Mails: gferesin@unsj.edu.ar (G.E.F.); blima.unsj@gmail.com (B.L.)

**Keywords:** propolis, antimicrobial, phenolics, flavonoids, diarylheptanoids, poilaneic acid

## Abstract

Propolis is commercialized in Chile as an antimicrobial agent. It is obtained mainly from central and southern Chile, but is used for the same purposes regardless of its origin. To compare the antimicrobial effect, the total phenolic (TP), the total flavonoid (TF) content and the phenolic composition, 19 samples were collected in the main production centers in the Región del Maule, Chile. Samples were extracted with MeOH and assessed for antimicrobial activity against Gram (+) and Gram (−) bacteria. TP and TF content, antioxidant activity by the DPPH, FRAP and TEAC methods were also determined. Sample composition was assessed by HPLD-DAD-ESI-MS/MS. Differential compounds in the samples were isolated and characterized. The antimicrobial effect of the samples showed MICs ranging from 31.5 to > 1000 µg/mL. Propolis from the central valley was more effective as antibacterial than those from the coastal area or Andean slopes. The samples considered of interest (MIC ≤ 62.5 µg/mL) showed effect on *Escherichia coli*, *Pseudomonas* sp., *Yersinia enterocolitica* and *Salmonella enteritidis*. Two new diarylheptanoids, a diterpene, the flavonoids pinocembrin and chrysin were isolated and elucidated by spectroscopic and spectrometric means. Some 29 compounds were dereplicated by HPLC-MS and tentatively identified, including nine flavones/flavonol derivatives, one flavanone, eight dihydroflavonols and nine phenyl-propanoids. Propolis from the Región del Maule showed large variation in antimicrobial effect, antioxidant activity and composition. So far the presence of diarylheptanoids in samples from the coastal area of central Chile can be considered as a marker of a new type of propolis.

## 1. Introduction

Propolis is a natural product made from a mixture of resinous substances collected by honeybees (*Apis mellifera*) from buds, bark and plant exudates. Its chemical composition is highly variable depending on the collection site, flora, and climate [1]. The demand for propolis is increasing due to its health benefits and use in cosmetic and food products. Chemical differences can be found in propolis according to the production areas. This chemical diversity is related to the bioactivity and potential uses of this product. Propolis is used in Chile for a variety of purposes, including to relieve sore throats, to prevent and alleviate cold symptoms, and as an antiseptic and anti-inflammatory. The product is used in alcoholic solutions, in candies, tablets, sprays and syrups. The concentration of propolis in over-the-counter preparations is variable, from 120 mg of propolis per tablet to 600 mg propolis in 15 mL of syrup. General recommendations indicate a three times per day dose, independent of therapeutic use. In rural areas of the Región del Maule, central Chile, apiculture is a relevant occupation. However, according to beekeepers from this region, only 1% of them are engaged in propolis production. This low percent is mainly due to the lack of knowledge about the beneficial health properties of propolis, poor chemical characterization, and little information about the bioactivity of the regional product.

**Figure 1:**
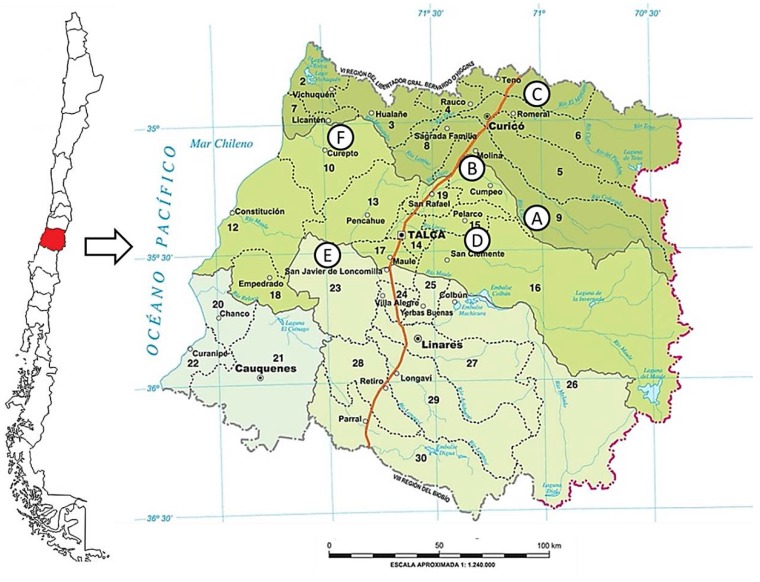
Map of Chile showing the location of the Región del Maule and the propolis collection places. Vilches (**A**); Cumpeo (**B**); Romeral (**C**); San Clemente (**D**); San Javier (**E**); Curepto (**F**).

Research on Chilean propolis has been focused so far on samples from Rincon de Yaquil [2,3], Cuncumen [4], Colliguay [5], Región de la Araucanía [6] and Región Metropolitana [7,8]. The mentioned studies reported mainly flavonoids, but lignans, coumarins and other constituents were also found [2], suggesting a broader chemical diversity in Chilean samples. A previous study on propolis from San Clemente, Región del Maule, reported six flavonoids including the flavanones/flavanols: 5,7-dihydroxyflavanone, 5-hydroxy-7-methoxyflavanone and 5,7-dihydroxyflavanol, as well as the three flavones: 5,7-dihydroxyflavone, 5-hydroxy-7-methoxyflavone and 3,5,7-trihydroxyflavone [9]. The Región del Maule in central Chile (35°58ʹ S, 70°38ʹ W) is characterized by three geomorphological zones, ranging from the Pacific ocean to the western Andean slopes (Figure 1). The climate is Mediterranean-like, with some variations due to increasing latitude and altitude. The sclerophyllous forest of the coastal areas, the cultivated central valley, and the Andean *Nothofagus* forests offer different sources of resins and nectar for bees. However, all propolis, regardless of their origin, are used for the same therapeutic purposes, often without any scientific validation. Hence the aim of the present work was to assess the antimicrobial effect, antioxidant capacity, and chemical constituents of propolis from the main production areas within the Región del Maule, central Chile. The samples can be considered representative of the propolis from the central part of the country.

## 2. Results and Discussion

### 2.1. Antibacterial Activity

The MeOH extracts from 19 propolis samples produced in the Región del Maule were assessed for antimicrobial effect against Gram (+) and Gram (−) bacteria of clinical importance (11 bacterial strains). The microorganism selection was based on the fact that propolis is topically used to treat skin infections, and therefore we included the Gram positive *Staphylococcus aureus*. Propolis is also used by oral administration to relieve gastrointestinal infections so several Gram negative enterobacteria were included. The results are presented as MIC values in µg/mL, allowing a comparison of the different samples according to the production location (Table 1).

Interesting differences were observed in the antibacterial effect of the samples. MICs values ranged from 31.2 to > 1000 µg/mL depending on the microorganisms and collection place. Among the different propolis extracts, propolis from the central valley was more effective as *in vitro* antibacterial agents than those from the coastal area or Andean slopes (MICs values between 62.5 and 1000 µg/mL). Most of the samples showed activity against *Pseudomonas* sp., *Yersinia enterocolitica*, and *Salmonella enteritidis* (MIC ≤ 62.5 µg/mL). From the central valley (San Clemente), four out of the five samples were active against *Escherichia coli* ATCC 25922 (MICs ≤ 62.5 µg/mL); three of the five were active towards *Y. enterocolitica*-PI and *S. enteritidis*. One of the samples (San Clemente 3) was active against 7 of the 11 microbial strains, with MICs of 62.5 µg/mL. However, the sample San Clemente 5 performed poorly against one of the microorganisms and was inactive against 10 other bacteria. This suggests that the origin of the sample does not necessary implies strong antimicrobial activity. In regards to the coastal area (San Javier), one of the samples (San Javier 5) presented activity against methicillin-resistant *S. aureus*, *E. coli*, *Pseudomonas* sp. and *S. enteritidis* with MICs ≤ 62.5 µg/mL. Overall, the propolis samples: Romeral 1, Cumpeo, San Clemente 2 and San Javier 5 showed the highest antibacterial activity against *E. coli* ATCC 25922 with MICs of 31.2 µg/mL.

Propolis extracts were evaluated for antimicrobial activity in several countries. The ethanol extract from Mexican propolis presented minimal bactericidal concentrations (MBC) in the range of 0.93–5 mg/mL for Gram (+) and 7.5–10 mg/mL for Gram (−) bacteria [10]. Colombian propolis showed MBC values that ranged from 15.39 to 17.03 mg/mL against *S. aureus* and from 17.03 to 30.78 mg/mL for *Pseudomonas aeruginosa* [11]. Propolis collected in Portugal, Silva *et al*. [12] reported MIC values (in µg/mL) ranging from 590 to 1720 for *S*. *aureus*, 1560 to 2810 for *P. aeruginosa* and 3190 to 4860 for *E. coli*. Campos *et al*. [13] found antimicrobial activity in an 80% ethanol extract of Brazilian stingless bees with MICs of 3100 µg/mL for *S. aureus* and *C. albicans*. The activity of these samples were very weak according to criteria requesting MIC values < 200 µg/mL for potential sources of antimicrobial agents. Rios and Recio [14] proposed guidelines for extracts and isolated compounds in order to find antimicrobial agents. The authors pointed out that an extract can be considered as promising if the activity (as MIC values) was <100 µg/mL. In a screening of antibacterial activity of propolis from Tucumán, Argentina, Moreno *et al*. [15] used the agar diffusion technique and found MIC values (in µg/mL) of 15.3–49.1 for *S. aureus*, 7.8–107.9 for *Streptococcus piogenes*, 7.5–77.1 for *Streptococcus agalactiae* and 14.0–210.0 for *Enterococcus faecalis*. For Chilean propolis collected in the Region de O’Higgins and the Region Metropolitana (north of the Region del Maule), the MIC values for the extracts were > 128 µg/mL against *Mycobacterium avium* ATCC 2591 and *M. tuberculosis* ATCC 27294 measured by the agar diffusion technique [3]. No effect by the disk diffusion assay at 500 µg/disk was found for *Enterococcus faecium* VREF and two *E. coli* strains [3]. An antibacterial effect by the microdilution assay was reported in a monofloral honey from Chile [16]. The antimicrobial assay was carried out with the phenolic-enriched honey extract and the MIC values (in µg/mL) were in the range of 1260–1350 for *Pseudomonas aeruginosa*, 320–680 for *Staphylococcus aureus*, 1260–1350 for *Salmonella typhi*, 340–1260 for *Streptococcus pneumonia* β-type and 1350 for *Vibrio cholera*. The phenolic-enriched extract was inactive against *E. coli* and the yeast *Candida albicans*.

The antibacterial activity of the samples was compared with the TP and TF content, as well as with the chromatographic profiles to look for some clues to better associate the activity with chemical constituents of propolis. The most and less active samples from the same geographic areas were selected for comparison according to the MIC values and overall antimicrobial effect. The samples were Romeral 1 and 5, San Clemente 3 and 5, San Javier 5 and 2. Romeral 1 was the most active sample. It contained higher TP and TF (20.15 g GAE (gallic acid equivalents)/100 g extract and 13.95 g CE (catechin equivalents)/100 g extract) than sample 5, which contained TP and TF of 12.16 g GAE/100 g extract and 5.01 g CE/100 g extract, respectively. The TP and TF content of the San Clemente 3 and San Clemente 5 samples were similar in TP (19.79 and 18.11 g GAE/100 g extract) and different in TF (7.52 and 3.67 g CE/100 g extract). However, this trend was not always related with antimicrobial effect, for example low TF containing sample San Clemente 4 (3.67 g CE/100 g extract) was quite active. The TP for the less active sample 2 and the most active sample 5 were 11.49 and 19.83 g GAE/100 g extract and TF were 4.96 and 10.34 g CE/100 g extract, respectively.

#### Antimicrobial Activity and Propolis Fingerprint

**Figure 2:**
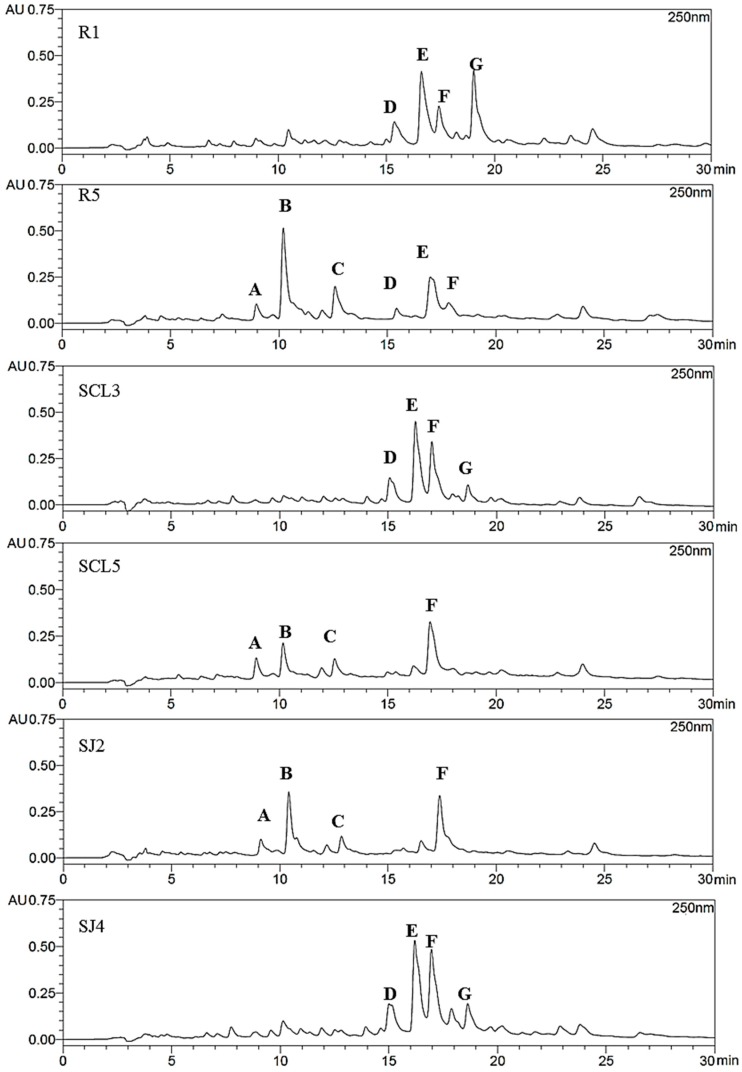
HPLC chromatograms of the most and less active antimicrobial propolis samples from the Región del Maule, Chile. Andean slopes: R1: Romeral 1; R5: Romeral 5; Central valley: SCL3: San Clemente 3; SCL5: San Clemente 5; Coastal area: SJ: San Javier 2; SJ4: San Javier 4. Detection: UV, 250 nm. A: flavonol; B: simple phenolic; C: caffeic acid derivative; D: flavone and dihydroflavonol; F: flavonol; G: phenylpropanoid.

**Figure 3:**
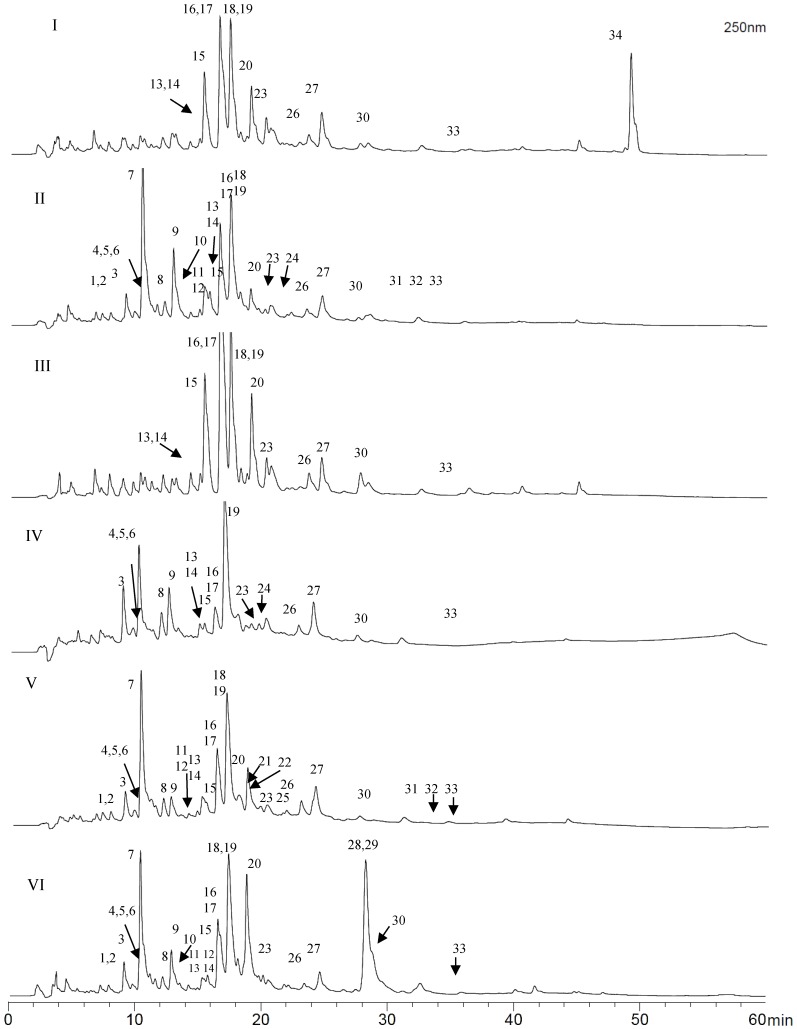
HPLC chromatograms of propolis from the Región del Maule, Chile, and tentative identification of phenolic compounds. Andean slopes: I: Vilches, II: Romeral 3; Central valley: III: Cumpeo, IV: San Clemente 5; Coastal area: V: San Javier 3; VI: Curepto 1. Detection: UV, 250 nm. For compound identification see Table 3.

The phenolic profiles from the most and less active propolis samples from the different geographic areas of the Región del Maule were compared by HPLC-DAD. The chromatograms are shown in Figure 2. The less active samples showed higher proportion of the polar constituents eluting at Rt 8.5–13 min (peaks A–C). The most active samples from the three floristic formations (R1, SCL3, SJ5) have a main set of constituents in common that elude at Rt 15–20 min (peaks D–G) but differ in the relative intensity/ratio of the compounds. The UV spectra of the compounds A, B and C shows maxima compatible with a flavonol (A), a simple phenolic (B) and a caffeic acid derivative (C). The peaks D, E, F and G, which are present in the most active samples of each production area, show UV maxima in accordance with caffeic acid derivatives (D), a flavone and dihydroflavone (E), a flavonol (F) and a phenylpropanoid different than caffeic acid (G). Thus, the antimicrobial activity could be associated with the occurrence and ratio of different constituents. When comparing all the samples from the Andean slopes, the propolis from Vilches (with moderate activity against 8 of the 11 bacteria) presented an additional peak at Rt 49.1 min (**34**). This compound showed an UV maxima of 249 nm and was less polar than the other constituents (Figure 3).

The propolis from Romeral 4, Vilches, San Clemente 3 and 4 and San Javier 4 and 5 are active against the *S. aureus* microorganism with MICs in the range 62.5 to 125 µg/mL. When considering the methicillin-resistant *S. aureus*, San Clemente 4 and San Javier 5 samples can be regarded as promising antibacterial agents with MICs of 62.5 µg/mL. In regards to the Gram (−) bacteria, *E. coli* was more susceptible to the Andean slopes and central valley samples, mainly against *E. coli* ATCC 25922 and the clinical isolate *E. coli* 122. When comparing the activity of propolis from Romeral against Gram (−) bacteria, Romeral 1 was the most interesting with MICs ≤ 125 µg/mL against 7 of the 9 bacteria. In the central valley samples the best antibacterial effect was from the Cumpeo propolis, with moderate to strong activity against 8 out of the 9 bacteria (Table 1).

**Table 1:** Antimicrobial activity of methanol extracts of propolis from the Andean slopes (“precordillera”), central valley, and coastal area (“secano costero”), Región del Maule, Chile. Results are presented as MIC values in µg/mL.

Propolis Sample	Microorganisms ^a^											
	Gram (+)						Gram (−)					
	1	2	3	4	5	6		7	8	9	10	11
Andean slopes												
Romeral 1	500	125	31.2	250	62.5	125		125	125	62.5	125	500
Romeral 2	250	125	62.5	250	62.5	125		62.5	125	62.5	250	>1000
Romeral3	500	250	125	250	250	250		250	250	250	250	500
Romeral 4	125	125	62.5	500	125	125		125	125	125	250	>1000
Romeral 5	1000	500	500	500	1000	500		500	250	500	500	>1000
Vilches	125	125	62.5	250	125	250		125	62.5	125	125	250
Central valley												
Cumpeo	250	125	31.2	125	62.5	62.5		62.5	62.5	62.5	125	250
San Clemente 1	500	250	125	1000	250	250		250	250	250	250	500
San Clemente 2	125	250	31.2	1000	125	125		125	62.5	62.5	250	500
San Clemente 3	62.5	125	62.5	250	62.5	62.5		62.5	250	62.5	62.5	500
San Clemente 4	62.5	62.5	62.5	1000	125	250		125	62.5	125	125	250
San Clemente 5	500	125	1000	>1000	>1000	1000		500	250	>1000	500	1000
Coastal area												
Curepto 1	125	125	125	500	125	250		125	125	125	125	500
Curepto 2	62.5	125	125	500	125	250		250	125	500	500	500
San Javier 1	125	250	62.5	>1000	125	125		250	125	125	250	>1000
San Javier 2	>1000	>1000	62.5	>1000	500	500		1000	500	>1000	500	1000
San Javier 3	500	250	125	250	250	250		250	250	250	250	500
San Javier 4	62.5	125	62.5	1000	125	125		62.5	125	125	250	500
San Javier 5	125	62.5	31.2	250	125	125		62.5	125	62.5	500	500
Cefotaxime	0.5	0.5	0.5	5.0	0.5	0.5		7.5	0.5	12.5	0.5	0.05

^a^ **1**: methicillin-sensitive *Staphylococcus aureus* ATCC 25923; **2**: methicillin-resistant *S. aureus* ATCC 43300; **3**: *Escherichia coli* ATCC 25922; **4**: *E. coli* 121; **5**: *E. coli* 122; **6**: *E. coli* LM_2_; **7**: *Pseudomonas* sp.; **8**: *Yersinia enterocolítica-* PI; **9**: *Salmonella enteritidis* MI; **10**: *Salmonella sp* (LM); **11**: *Proteus mirabilis* 94-2.

### 2.2. Total Phenolic, Total Flavonoid Content and Antioxidant Activity

Some 19 propolis samples from the main apicultural areas of the Región del Maule, central Chile, were investigated for percent extraction yield in methanol, total phenolic, total flavonoid content, antioxidant activity and phenolic composition (Table 2). Extraction yields with methanol were high, with values (%) of 76.85 (Cumpeo), 54.38 and 55.84 (Curepto), 51.76 and 67.68 (Romeral), 61.11 to 78.89 (San Javier) and 58.85–78.65 (San Clemente). Most of the samples presented TP values in the range of 14.69–20.84 g GAE/100 g, except Romeral 5 (12.16 g GAE/100 g) and San Javier 2 (11.49 g GAE/100 g). The TF content was variable, with seven samples presenting low values ranging from 1.72–5.03 g CE/100 g and 12 collections with high values ranging from 7.52–14.03 g CE/100 g.

The *in vitro* antioxidant capacity of the samples was evaluated by the DPPH, TEAC and FRAP assays. Results are summarized in Table 2. The most active samples in the DPPH assay, with SC_50_ values ranging from 10.29 to 24.68 µg/mL were those of Cumpeo (15.45 µg/mL), Vilches (24.68 µg/mL), omeral (16.16, 18.93 and 20.06 µg/mL), San Javier (10.29 and 24.11 µg/mL) and San Clemente (20.10 µg/mL). The activity is related with the TP content of the samples (r = 0.90, *p* < 0.05). In the FRAP assay there was no clear relation between TP and TF content but the samples with lower TP and TF content were the least active in the FRAP assay (Table 2). In the TEAC determination, in a broad sense, the activity can be related with the TP content. The most active antioxidant propolis samples were those from the western Andes slopes (“precordillera”). This area is characterized by the co-existence of native flora, including *Nothofagus* trees, fruit orchards, pine trees and *Eucalyptus* plantations. In the central valley, the vegetation is similar to the Mediterranean region of Europe with large quantities of agricultural crops and fruit orchards. The coastal area known as “secano costero” or “matorral costero” contains native shrubs and trees that have adapted to dry conditions, as well as some agricultural crops and tree farms [2,3]. The variability in the antioxidant activity can be attributed to the composition of the samples, which is related to the botanical sources of propolis. Flavonoids and phenolic acid derivatives may be responsible for the antioxidant activity of propolis.

### 2.3. Isolation of New Propolis Constituents

The QTOF-MS of compound **28** and **29** presented ions at *m*/*z* 363.1588, 347.1814 and 247.1697, in agreement with the molecular formula C_21_H_24_O_3_K, C_21_H_24_O_3_Na, and [M]^+^-acetate-water. The IR spectrum indicated the presence of a hydroxyl, a carbonyl function and a α,β unsaturated ester according to the band at 3450, 1732 and 1238 cm^−1^, respectively. The ^1^H-NMR spectrum of both compounds showed a signal at 7.02–7.25 *m* for two aromatic mono-substituted rings, a pair of *trans* olefinic protons at δ 6.48–6.49 (d, 16 Hz) and 6.05–6.07 (dd, 16; 6.8 Hz), but differs in the ester and hydroxyl protons at δ 5.04 and 4.11 for **28** and δ 5.57 as well as δ 3.48 for **29**. A methyl group δ 1.94 for compound **28** and δ 1.97 for compound **29** was connected to a carbonyl group at δ 172.15 indicating the presence of acetate in both compounds. This observation was confirmed by HMBC experiments. HMBC analysis showed that the difference in both compounds was the placement of the acetate, being at C-5 for **28** and C-3 for **29**. The structures were established as (*E*)-3-hydroxy-1,7-diphenylhept-1-ene-5-acetate (**28**) and (*E*)-5-hydroxy-1,7-diphenylhept-1-ene-3-acetate (**29**).

The isolated compounds pinocembrin (**17**) and chrysin (**16**) were also evaluated as antimicrobial agents showing MICs > 50 µg/mL against methicillin-sensitive *Staphylococcus aureus* ATCC 25923, methicillin-resistant *S. aureus* ATCC 43300, *Escherichia coli* ATCC 25922, *E. coli* 121, *E. coli* 122, *E. coli* LM_2_, *Salmonella* sp. LM and *Proteus mirabilis* 94-2. While the MIC of pinocembrin against *Pseudomonas* sp., *Yersinia enterocolítica* PI and *Salmonella enteritidis* MI was 50 µg/mL, the MIC values for chrysin were > 50 µg/mL (data not shown).The isolated compounds poilaneic acid (**34**), and the diarylheptanoides **28** and **29** were not evaluated as antimicrobial agents due to the low amount of the compound available.

In propolis from Colliguay, Central Chile (33° S 71° W), a diarylheptanoid (*trans*-3,5-dihydroxy-1,7-diphenyl-hept-1-ene), flavonoids and coumarins were reported [5]. However, the diarylheptanoid described was a diol, while the sample from Curepto showed a mixture of the diarylheptanoid monoacetates **28** and **29**. The compound might have a common botanical source, as both locations are placed in the coastal area and the vegetation is similar. The sample from Curepto containing the diarylheptanoids was provided by beekeepers from Rapilermo Alto, a small village in the coastal area. The sample was referred as “from Curepto” due to the beekeepers production area. In 2001, Muñoz *et al*. [5] reported the isolation of a diarylheptanoid from a propolis sample from Colliguay, a small village located in the coastal area of central Chile. Combining this report and our findings, we can now suggest that diarylheptanoids might be used to differentiate a new type of propolis occurring in the coastal area of central Chile. Diarylheptanoids were reported from the Asian endemic species *Alpinia katsumadai* [17,18,19,20], *Alpinia officinarum* [21,22,23] and *Alnus nepalensis* [24], among others. Most of the diarylheptanoids reported have substituents in the aromatic ring [25].

**Figure 4:**
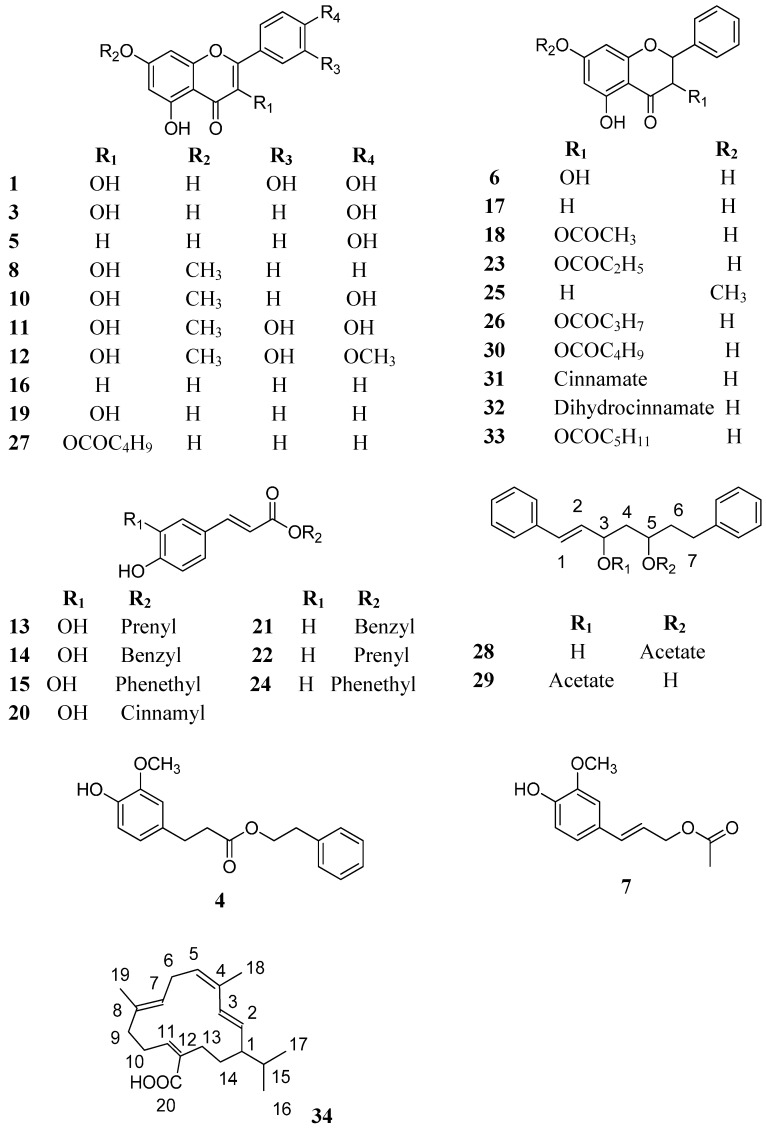
Structure of the compounds identified/tentatively identified in propolis from the Región del Maule, central Chile.

Compound **34** was obtained as a colorless gum. The IR spectrum indicated the presence of a hydroxy and a conjugated carboxylic acid function at 3413 and 1634 cm^−1^, respectively. In the ^1^H-NMR spectrum of **34**, two conjugated and one isolated double bonds, an α,β-unsaturated carboxylic acid function, an isopropyl group and two olefinic methyl groups were observed. The ^13^C-NMR spectrum showed 20 C signals, including a conjugated carboxylic acid at δ 172.83 ppm, four double bonds, five CH_2_ groups, an isopropyl and two methyl groups associated to the double bonds, suggesting a cyclic compound. The QTOF-mass spectrum showed a pseudomolecular ion at *m*/*z* 341.2256 amu, in agreement with a molecular formula C_20_H_30_O_2_K and an unsaturation index of 6, pointing out to a macrocyclic diterpene. Extensive NMR analysis allowed the identification of **34** as the cembrane diterpene poilaneic acid (Figure 4). This is the first report about its occurrence in propolis. The ^1^H-NMR and ^13^C-NMR data are in agreement with [26]. Poilaneic acid was previously reported from the Asian Euphorbiaceae *Croton poilanei* [26] and *Croton robustus* [27]. However, in [27], the C atoms of the compound were wrongly assigned. The weak inhibitory activity of poilaneic acid on cAMP phosphodiesterase was described [28]. This work reports for first time the occurrence of the compounds **28**, **29** and **34** in propolis.

**Table 2:** Extraction yields, total phenolics (TP), total flavonoids (TF) and antioxidant activity of methanol extract of propolis from the Andean slopes (“precordillera”), central valley and coastal area (“secano costero”), Región del Maule, Chile.

Propolis Sample	% (w/w) Extraction Yield	Total Phenolics (g Gallic Acid Equivalents/100 g MeOH Extract)	Total Flavonoids (g Catechin Equivalents/100g MeOH Extract)	DPPH (SC_50_ in µg/mL or % Inhibition at 100 µg/mL)	FRAP (µmol Trolox Equivalents/g MeOH Extract)	TEAC (µM Trolox Equivalents/g MeOH Extract)
Andean slopes						
Romeral 1	49.60	20.15 ± 0.71	13.95 ± 0.65	16.16 ± 1.87	1093.91 ± 63.85	1968.20
Romeral 2	43.21	17.29 ± 0.96	11.09 ± 1.92	20.06 ± 0.80	866.81 ± 47.71	1876.35
Romeral3	67.68	17.78 ± 1.04	8.80 ± 0.55	42.68 ± 0.65	1133.45 ± 51.81	2216.85
Romeral 4	43.86	20.69 ± 0.15	14.03 ± 1.57	18.93 ± 1.00	1066.18 ± 52.19	2328.66
Romeral 5	51.76	12.16 ± 0.41	5.01 ± 0.20	86.94 ± 1.47	806.87 ±35.16	1718.38
Vilches	49.36	18.27 ± 0.86	11.01 ± 0.61	24.68 ± 1.84	843.16 ± 55.49	2230.17
Central valley						
Cumpeo	76.85	20.84 ± 0.55	13.27 ±1.94	15.45 ± 0.89	958.95 ± 44.39	2000.37
San Clemente 1	67.21	16.72 ± 1.72	4.65 ± 0.17	51.51 ± 1.47	1032.34 ± 36.04	1730.21
San Clemente 2	78.13	17.34 ± 1.10	9.86 ± 0.51	20.10 ± 1.18	1029.89 ± 47.67	1583.85
San Clemente 3	58.85	19.79 ± 0.44	7.52 ± 0.70	Inactive	Inactive	1347.61
San Clemente 4	77.27	20.11 ± 0.75	1.72 ± 0.21	24.82% ± 0.91%	Inactive	1922.57
San Clemente 5	78.65	18.11 ± 0.21	3.67 ± 0.20	58.51 ± 1.50	1151.39 ± 65.19	2136.81
Coastal area						
Curepto 1	54.38	15.28 ± 0.03	4.60 ± 0.20	71.65 ± 1.67	742.05 ± 33.93	2107.04
Curepto 2	55.84	18.75 ± 0.82	9.33 ± 0.48	29.70 ± 1.01	1101.65 ± 52.98	2212.13
San Javier 1	47.57	14.69 ± 0.29	5.03 ± 0.06	70.90 ± 1.61	810.13 ± 31.29	1763.19
San Javier 2	39.53	11.49 ± 0.05	4.96 ± 0.43	91.84 ± 1.63	667.43 ± 42.38	870.64
San Javier 3	78.89	18.51 ± 0.65	8.07 ± 0.58	31.13 ± 0.97	1241.91± 46.71	2146.35
San Javier 4	69.21	19.62 ± 0.72	9.60 ± 1.30	10.29 ± 1.17	1745.03± 124.41	1606.90
San Javier 5	61.11	19.83 ± 0.66	10.34 ± 1.09	24.11 ± 2.42	836.23 ± 15.49	1745.33
Quercetin				7.82 ± 0.30	10769.85 ± 164.33	8157.90

Determinations of TP, TF, DPPH and FRAP were performed in triplicate and results are expressed as mean values ± SD. For the TEAC assay, a curve was plotted for each sample and a correlation coefficient with a 95% confidence limit was established.

### 2.4. Identification of Phenolics in Propolis from the Región del Maule

The identification of phenolic constituents in propolis from the Región del Maule was carried out using a combination of chromatographic, spectroscopic and spectrometric methods. The data was analysed and compared with previously published reports. The main constituents of propolis were isolated by chromatography and were identified by NMR spectroscopy, UV and MS. The fully characterized compounds were used as markers for comparison and their MS fragmentation afforded valuable information for a comparative analysis using HPLC-DAD-MS/MS techniques.

All propolis samples were compared by HPLC-DAD to obtain profiles from the different collection/production areas. Selected HPLC chromatograms are shown in Figure 3. The compounds tentatively identified in the collections are summarized in Table 3 and the structures are shown in Figure 4. The UV spectra and MS fragmentation patterns were compared with literature for assignation [29,30,31,32,33,34]. A total of 34 compounds were identified in the samples, including 20 flavonoids, nine phenylpropanoid esters, two diarylheptanoids, a diterpene, ellagic acid methyl ether and a caffeic acid derivative.

The flavonoids included eight flavonols and its esters (compounds **1**, **3**, **8**, **10**, **11**, **12**, **19** and **27**), eight dihydroflavonols and its esters (compounds **6**, **18**, **23**, **26**, **30**–**33**), two flavones (compounds **5** and **16**) and two flavanones (compounds **17** and **25**). The phenylpropanoids included four caffeic acid esters (**13**–**15** and **20**), three *p*-coumaric acid esters (**21**, **22** and **24**), coniferyl acetate (7) and dihydroferulic acid phenethyl ester (**4**). The main compounds were the flavone chrysin **16**, the flavanone pinocembrin (**17**), the dihydroflavonol pinobanksin acetate (**18**), the flavonol galangin (**19**) and the caffeic acid esters **13**–**15** and **20** occurring in most samples. Coniferyl acetate (**7**) was found in several samples, including propolis from the coastal area (both from Curepto and three out of five samples from San Javier), two out of five samples of the Andean slopes (Romeral) and two out of five collections from the central valley (San Clemente). The chemical composition of propolis from the Región del Maule is similar to propolis samples from Europe [32,33,35], South Africa [36] and China [37,38]. Chrysin (**16**), pinocembrin (**17**) and the pinobanksin esters (**18**, **23**, **26**, **30**, **33**), as well as the caffeic acid esters **14**–**15** have been found to be main constituents in propolis from the Región del Maule in central Chile, as well as for samples from Europe and China. The similarity in constituents might be due to some introduced species, including *Populus*, *Eucalyptus*, *Pinus*, as well as crops. In Europe *Populus* is considered to be the main botanical source of propolis [1].

The compounds **16**–**18**, **23**, **26**, **30** and **33** were also described in propolis from Argentina [39,40] and Uruguay [41]. Propolis from others regions of Chile showed differences with the samples from the Región del Maule. In samples from the Región de la Araucanía, mainly pinocembrin (**17**) and CAPE (**15**) were reported [6], while propolis from Santiago has a composition similar to the propolis from the Región del Maule [7]. The presence of chrysin (**16**), galangin (**19**) and pinocembrin (**17**) in propolis from the Andean slopes of the Región del Maule has been previously described [9]. Compound **7** was previously isolated from the Asteraceae *Olearia teretifolia* and identified as coniferyl 9-acetate [42]. Coniferyl acetate was reported in propolis from Cruce de Yaquil (Región de O’Higgins, Chile) [3].

The antioxidant activity of various propolis was assessed using the DPPH assay [35]. Samples with higher phenolic content had stronger activity compared to those with other types of constituents. Caffeic acid, kaempferol, CAPE and cinnamyl caffeate favored the antioxidant capacity [35]. Propolis from the Región del Maule containing chrysin, galangin and pinocembrin exhibited a strong effect as a scavenger of the DPPH radical [9]. These results are in agreement with those reporting the antioxidant activity of galangin- and pinocembrin-rich propolis from India, which showed excellent antioxidant activities [43]. In agreement with previous reports, the antioxidant activity in Chinese propolis was evaluated using DPPH, FRAP and ABTS assays [38]. Galangin exhibited strong activity in two of the assays, while caffeic acid derivatives (CAPE, cinnamyl caffeate and benzyl caffeate) showed strong activity in all antioxidant assays. Therefore, the results on the antioxidant activity in propolis from the Región del Maule can be associated with the presence of phenolics, mainly compounds **15**, **17**, **19** and **20**. The diarylheptanoids **28** and **29** can be considered so far as marker compounds for propolis from Curepto, a place located in the coastal area of central Chile, while the diterpene **34** appear to be characteristic of the western Andean slopes.

## 3. Experimental Section

### 3.1. Propolis Samples: Geographic Origin and Extraction

Nineteen raw propolis samples were provided by beekeepers from the Región del Maule. All samples investigated were from single apiaries and were not mixed. Therefore, they represent the composition of propolis from single producers from the same area. The producers were selected on the basis of the main providers of apicultural products in the region. The samples were collected in years 2012–2014 and were frozen at −20 °C before processing. The collection places were as follows: Vilches (35°29ʹ S, 71°08ʹ W; 1 sample, 2012), Cumpeo (35°16ʹ S, 71°15ʹ W; 1 sample, 2012), Romeral (34°57ʹ S, 71°07ʹ W; 5 samples, 2014), San Clemente (35°33ʹ S, 71°29ʹ W; 5 samples, 1 from 2013, 4 from 2014), San Javier (35°59ʹ S, 71°44ʹ W; 5 samples, 2014) and Curepto (35°05ʹ S, 72°01ʹ W; 2 samples, 2014). The different samples from the same geographic area belong to different apiaries. They represent the different sources of plant material for propolis production. The collection places are shown in Figure 1. Approximately ten grams of each propolis sample was extracted at room temperature two times with 100 mL MeOH, sonicated during 3 min and filtered. The remaining solid was re-extracted two times with 50 mL MeOH, filtered, and the combined MeOH solubles were concentrated under reduced pressure to afford the corresponding crude MeOH extract. Extracts were stored in the dark at −20°C until analysis.

### 3.2. Chemicals

Folin-Ciocalteu phenol reagent, 2,4,6-tri(2-piridyl)-1,3,5-triazine sodium acetate (TPTZ), 1,1-diphenyl-2-picrylhydrazyl radical (DPPH), quercetin, catechin, gallic acid and AlCl_3_ were purchased from Sigma-Aldrich (St. Louis, MO, USA), 2,2ʹ-azino-bis(3-ethylbenzothiazoline-6-sulfonic acid diammonium salt (ABTS), 6-hydroxy-2,5,7,8-tetramethylchroman-2-carboxylic acid (Trolox) from Calbiochem (San Diego, CA, USA). HPLC-grade methanol, formic acid and thin layer chromatography plates (TLC, Kieselgel F254), were purchased from Merck (Darmstadt, Germany). Ultrapure water was obtained using a Barnsted EasyPure water filter (Thermo Scientific, Marietta, OH, USA).

**Table 3:** Tentative identification of phenolic compounds in propolis from the Región del Maule by HPLC-ESI-MS/MS.

Compound	Rt (min)	UV Max	[M − H]^−^	MS/MS	Tentative Identification
**1**	7.5	-	301	178, 150	Quercetin ^(a,b)^
**2**	8.0	-	315	300	Ellagic acid methyl ether ^(a)^
**3**	9.2	363, 256	285	257, 241, 229, 168, 150	Kaempferol ^(a,b)^
**4**	9.4	-	299	284, 178, 134	Dihydroferulic acid phenethyl ester ^(a)^
**5**	10.0	-	269	225, 150	Apigenin ^(a,b)^
**6**	10.2	-	271	253, 225, 215, 197, 150	Pinobanksin ^(a,b)^
**7**	10.5	302 sh, 266	221		Coniferyl acetate ^(c)^
**8**	12.0	349 sh, 301 sh, 267	283	268, 239, 211	Galangin methyl ether ^(a,b)^
**9**	12.5	322, 293 sh	415	371, 315, 178, 134	Caffeic acid derivative ^(a)^
**10**	12.8	-	299	284	Kaempferol methyl ether ^(a,b)^
**11**	13.4	-	315	300, 192, 165	Rhamnetin (Q-methyl ether) ^(a,b)^
**12**	13.4	-	329	314	Quercetin dimethyl ether ^(a,b)^
**13**	15.0	326, 297 sh	247	178, 135	Caffeic acid prenyl ester ^(a,b)^
**14**	15.0	326, 297 sh	269	225, 177, 133	Caffeic acid benzyl ester ^(a)^
**15**	15.5	330, 297 sh	283	178, 135	Caffeic acid phenylethyl ester ^(a,b)^
**16**	16.6	310, 268	253	209, 151	Chrysin ^(a,c)^
**17**	16.8	334 sh, 280	255	213, 150	Pinocembrin ^(a,c)^
**18**	17.5	288	313	253	Pinobanksin acetate ^(a,c)^
**19**	17.5	357, 268	269	241, 227, 197, 166	Galangin ^(a,b)^
**20**	19.0	323, 295 sh	295	251, 211, 177, 133	Caffeic acid cinnamyl ester ^(a)^
**21**	19.2	-	253	209, 161, 118	*p*-Coumaric acid benzyl ester ^(a)^
**22**	19.7	-	231	187, 161, 118	*p*-Coumaric acid prenyl ester ^(a)^
**23**	20.0	291	327	271, 253	Pinobanksin propionate ^(a,b)^
**24**	20.5	-	267	162, 118	*p*-Coumaric acid phenetyl ester ^(a)^
**25**	22.3	-	269	254, 236, 226,165	Pinocembrin methyl ether ^(a,b)^
**26**	23.6	-	341	253	Pinobanksin butyrate ^(a,b)^
**27**	24.5	-	353	253	Galangin pentanoate ^(a)^
**28**	28.3	251	323		3-hydroxy-1,7-diphenylhept-1-ene-5-acetate^(c)^
**29**	28.3	251	323		5-hydroxy-1,7-diphenylhept-1-ene-3-acetate^(c)^
**30**	28.5	-	355	253	Pinobanksin pentanoate ^(a,b)^
**31**	31.4	-	401	271, 253	Pinobanksin cinnamate ^(a)^
**32**	33.5	-	403	271, 253	Pinobanksin dihydrocinnamate ^(a)^
**33**	35.5	-	369	271, 253	Pinobanksin hexanoate ^(a,b)^
**34**	49.1	249	301	257	Poilaneic acid ^(c)^

Identification according to: ^a^ Confirmed by fragmentation pattern; ^b^ Confirmed by reference [29,30,31,32,33,34]; ^c^ Identified by NMR analysis; sh: shoulder.

### 3.3. Equipment

Nuclear magnetic resonance (NMR) spectra were recorded on a Bruker Avance 400 (Bruker, Rheinstetten, Germany) spectrometer at 400 MHz for ^1^H and 100 MHz for ^13^C in CDCl_3_ or MeOH-*d_4_*. Chemical shifts are given in ppm and coupling constant in Hz. QTOF-MS experiments were carried out using a Micromass Q-Tof micro (Waters, Manchester, UK). FT-IR was performed using a Nicolet Nexus 470 FT-IR (Thermo Electron Corporation, Waltham, MA, USA).

### 3.4. Antibacterial Activity 

#### 3.4.1. Microorganisms

The antibacterial activity of the extracts and compounds was assessed against the following bacteria: Gram (+): methicillin-sensitive *Staphylococcus aureus* ATCC 25923, methicillin-resistant *Staphylococcus aureus* ATCC 43300, and Gram (−): *Escherichia coli* ATCC 25922, and the clinical isolated *Escherichia coli*-121, *E. coli* 122 (Laboratorio Hospital Marcial Quiroga, San Juan, Argentina), *E. coli* LM2 (LM: Laboratorio de Microbiología, Facultad de Ciencias Médicas, Universidad Nacional de Cuyo, Mendoza, Argentina), *Salmonella enteritidis* MI (MI-Instituto Malbrán, Buenos Aires, Argentina), *Salmonella* sp (LM), *Yersinia enterocolítica*- PI (PI: Pasteur Institute, Buenos Aires, Argentina); *Pseudomonas* sp., *Proteus mirabilis* 94-2 (Laboratorio Hospital Marcial Quiroga, San Juan, Argentina). Bacteria were grown in Mueller Hinton broth medium.

#### 3.4.2. Antibacterial Activity of the Extracts

The MIC values were determined using the microbroth dilution method according to the protocols of the CLSI [44]. All tests were performed in Mueller Hinton broth (MHB), and cultures of each strain were prepared overnight. Microorganism suspensions were adjusted in a spectrophotometer with sterile physiological solution to give a final organism density of 0.5 McFarland scale (1–5 × 10^5^ CFU/mL). Stock solutions of extracts in DMSO were diluted to give serial two-fold dilutions that were added to each medium to obtain final concentrations ranging from 16–1000 µg/mL. The final concentration of DMSO in the assay did not exceed 1%. The antimicrobial agent Cefotaxime Argentia® (Bristol-Myers Squibb, Buenos Aires, Argentina) was included in the assays as a positive control. The plates were incubated for 24 h at 37 °C. Activity was evaluated at 620 nm using a Multiskan FC instrument. The MIC values were defined as the lowest extract/compound concentrations showing no bacterial growth after the incubation time. Tests were done in triplicate.

### 3.5. Total Phenolic (TP) and Flavonoid (TF) Content

The TP was determined by the Folin-Ciocalteu method [45]. Five mg of each methanolic extract were dissolved in 1 mL of MeOH. Then, 1 mL of each solution was mixed with the Folin-Ciocalteu reagent (0.2 mL) and after 5 min of reaction at room temperature 1 mL of warm Na_2_CO_3_ solution (20% w/v in bidestilated water) was added, and then completed with bidestilated water to a final reaction volume of 25 mL. Sixty minutes later the absorbance was measured at 725 nm. A calibration curve was performed with the standard gallic acid (0–120 mg/L, r = 0.9994). The results are expressed as g gallic acid equivalents/100 g MeOH extract of propolis. The TF content was determined according to previously described procedures [45,46]. Catechin was used as a reference standard and the TF was expressed as g catechin equivalents/100 g MeOH extract of propolis. All determinations were carried out in triplicate and are reported as mean values ± SD.

### 3.6. Antioxidant Activity Assays

#### 3.6.1. DPPH Assay

The free radical scavenging activity of the samples was determined by the DPPH assay according to [45]. Methanolic extracts were dissolved in 50% v/v aqueous MeOH to a final concentration of 300 μg/mL, filtered and kept in the dark. The stock solutions were serially diluted in 96-well microplates to final concentrations of 100, 33, 11 and 3 μg/mL. The DPPH solution was freshly prepared in methanol (20 mg/L) and 200 μL mixed with 100 μL of the extract at different concentrations. Methanol was used as the negative control, and quercetin was used as the positive control. The reaction mixture was incubated for 5 min at room temperature and absorbance was measured at 517 nm in a universal microplate reader (Biotek Instruments Inc., ELx 800, Winooski, VT, USA). The scavenging of DPPH radical was calculated as a percent of discoloration above the negative control group. SC_50_ values (μg/mL), corresponding to the extract amount that scavenges the radical concentration by 50%, were calculated from the dose-response curves using the OriginPro 8.0 software (OriginLab Corporation, Northampton, MA, USA). The determinations were performed in triplicate and are reported as mean values ± SD.

#### 3.6.2. FRAP (Ferric Reducing Antioxidant Power) Assay

The determination of ferric reducing antioxidant power or ferric reducing ability was performed as previously described [45,46,47]. The FRAP working solution was prepared mixing 300 mM acetate buffer, 10 mM TPTZ and 20 mM FeCl_3_ solution in a 10:1:1 v/v/v ratio. A 300 µg/mL extract aliquot (0.15 mL) was mixed with 2.85 mL of FRAP solution pre-warmed at 37 °C and left to stand in the dark for 30 min. Absorbance was measured at 593 nm using a Thermo Spectronic Helios Alfa spectrophotometer (Cambridge, UK). Quantification was performed using a standard curve of the antioxidant Trolox (from 60 to 480 μM; r = 0.9981). Samples were performed in triplicate and results were expressed as μmol Trolox equivalents (TE)/g extract. The determinations were performed in triplicate and are reported as mean values ± SD.

#### 3.6.3. TEAC (Trolox Equivalent Antioxidant Activity) Assay

ABTS radical-scavenging activity was determined by direct absorbance measurement of the radical (ABTS^+^) [48]. The radical was generated by mixing 5 mL of ABTS with 88 μL of 140 mM potassium persulfate. The solution was stored in the dark for 16 h, and diluted with methanol to a final absorbance of 0.700 ± 0.050 at 734 nm. The solution was used within the same day by mixing 2.87 mL of ABTS^+^ with 30 μL of fresh standard (1 mM Trolox) or extract (0–300 µg/mL). After 6 min, the absorbance was read at 734 nm, using methanol as blank. The values were obtained from the decrease in absorbance of radical ABTS^+^ at this defined point, and are expressed as μM Trolox/g extract.

### 3.7. HPLC-DAD Analysis

The composition of the propolis samples was analyzed by HPLC-DAD. The HPLC was a Shimadzu system (Shimadzu Corporation, Kyoto, Japan) consisting of a LC-20AT pump, a SPD-M20A UV diode array detector (DAD), CTO-20AC column oven and a LabSolution software. A MultoHigh 100 RP 18–5µ (250 × 4.6 mm) column (CS-Chromatographie Service GmbH, Langerwehe, Germany) maintained at 25 °C was used. Approximately 8 mg of each propolis extract was dissolved in 1.5 mL MeOH, filtered through a 0.45 µm PTFE (polytetrafluoroethylene) filter (Waters) and submitted to HPLC-DAD analysis. The compounds were monitored at 250 nm and 330 nm. UV spectra from 200 to 600 nm were recorded for peak characterization. The HPLC analysis was performed using a linear gradient consisting of 0.1% formic acid (A) and acetonitrile (B) as follows: 0–15 min, 40%–60% B; 15–30min, 60% B; 30–40 min, 60%–80% B; 40–50min, 80%–100%B; 50–60min, back to 40% B. The flow rate was 1 mL/min. The volume injected was 20 µL.

### 3.8. HPLC-ESI-MS/MS Analysis

The conditions of analysis were the same as for HPLC-DAD. HPLC-ESI-MS/MS data were recorded using a system which consisted of the HPLC HP1100 (Agilent Technologies Inc, CA, USA) connected through a split to the mass spectrometer Esquire 4000 Ion Trap LC/MS(n) system (Bruker Daltonik GmbH, Bremen, Germany). Ionization was performed at 3000 V assisted by nitrogen as nebulizing gas at 24 psi and as drying gas at 365°C and a flow rate of 6 L/min. Negative ions were detected using full scan mode (*m*/*z* 20–2200) and normal resolution (scan speed 10,300 *m*/*z*/s; peak with 0.6 FWHM/*m*/*z*). The trap parameters were set in ion charge control (ICC) using manufacturer default parameters, and maximum accumulation time of 200 ms. Collision induced dissociation (CID) was performed by collisions with helium background gas present in the trap and automatically controlled through SmartFrag option.

### 3.9. Isolation of Main Propolis Compounds

The extracts of all propolis samples were compared by HLPC-DAD. Most of the samples showed a similar pattern but some presented characteristic peaks that were considered differential compounds. Therefore, the main compounds were isolated by chromatographic means for full identification using spectroscopic and spectrometric means.

#### 3.9.1. High Speed Countercurrent Chromatography (HSCCC)

The isolation of diarylheptanoids from propolis extract was carried out using a Mk5 QuikPrep 500 HSCCC instrument (AECE, S. Wales, UK) equipped with four PTFE multilayer coil of 5 mm × 2.16 i.d. (SS) tubing of approximately 120 mL of volume capacity each. The solvent was pumped into the column with a SSi Serie II HPLC pump using a constant flow of 4 mL/min. A manual sample injection valve with 10 mL loop was used to introduce the sample into the column. The fraction collector used was a Gilson. Inc. (Middleton, WI, USA) model FC 203B. Several solvents systems were tested to find a suitable liquid-liquid separation system that allow to separate polar and medium polar compounds from propolis. The solvents system tested in this study were: petroleum ether (PE)/ethyl acetate/methanol/water (3:5:3:5), PE/ethyl acetate/methanol/water (4:5:4:3), PE/ethyl acetate/methanol/water (5:5:4:2) and PE/ethyl acetate/methanol/water (4:6:4:2). To choose the most suitable solvent system, the crude extracts were applied on silica gel and the TLC plates were developed with the upper phase and lower phase as the eluting solvent. Chromatograms were visualized under UV light (254 and 365 nm) and developed by spraying it with a methanolic solution of diphenyl boric acid ethanolamine complex (NPR). The biphasic solvent system composed of PE/ethyl acetate/methanol/water (5:5:4:2) was used for HSCCC separation. The solvent system was added to a separator funnel and thoroughly equilibrated at room temperature. The two phases were separated and sonicated prior to use. The upper phase was used as the mobile phase and the lower phase was used as the stationary phase in the “tail to head” mode. The multilayer coil column of the HSCCC was first filled entirely with the stationary phase. The mobile phase was then pumped into the tail end of the inlet column at a flow rate of 3.5 mL/min while the apparatus was rotated at 600 rpm. After the mobile phase front emerged and hydrodynamic equilibrium was established in the column, the crude propolis extract from Curepto (500 mg) dissolved in 4 mL each of upper and lower phase. Sample was injected via a glass syringe to a 10 mL sample loop. The HSCCC separation was performed at 20 °C. In the elution of the compounds, about 320 fractions of 3.5 mL each were collected. After stopping rotation, additional 320 fractions of 4 mL each were collected by extrusion. Fractions were monitored by TLC using the following mixture as the mobile phase: CHCl_3_/EtOAc (9:1). Those fractions with similar TLC pattern were combined and taken to dry under reduced pressure. The combined fractions 59–80 was purified by preparative TLC (silica gel, CHCl_3_:EtOAc 9:1 v/v), affording 18 mg of a mixture of two diarylheptanoids (Rf 0.63). The main compound was identified as 3-hydroxy-1,7-diphenylhept-1-ene-5-acetate **28** and the minor constituent as 5-hydroxy-1,7-diphenylhept-1-ene-3-acetate **29**. Fractions 202-239 (28 mg) afforded coniferyl acetate **7**, in agreement with the ^1^H-NMR and MS data [42].

*3-Hydroxy-1,7-diphenylhept-1-ene-5-acetate* (**28**). Pale yellow gum, UV (MeOH) λ_max_ 251 nm; IR (KBr) *v*_max_ 3450 (OH), 1732 (C=O ester), 1238 (ester) cm^−1^; ^1^H-NMR (400 MHz, CDCl_3_): δ 1.69 (2H, m, H-4), 1.75 (2H, m, H-6), 1.94 (3H, s, acetate-Me), 2.54 (2H, m, H-7), 4.11 (1H, m, H-3), 5.04 (1H, m, H-5), 6.07 (1H, dd, *J* = 16.0, 6.8 Hz, H-2), 6.48 (1H, d, *J* = 16.0 Hz, H-1), 7.02–7.25 (10H, m, H-2ʹ- H-6ʹ); ^13^C-NMR (100 MHz): δ 21.24 (CH_3_, CO*C*H_3_), 31.93 (CH_2_, C-7), 36.60 (CH_2_, C-6), 42.79 (CH_2_, C-4), 68.49 (CH, C-3), 71.25 (CH, C-5), 126.06 and 127.64 (CH, C-4’), 126.47 and 128.34 (CH, C2ʹ, 2C, C6ʹ, 2C), 128.51 and 128.58 (CH, C-3ʹ, 2C, C5ʹ, 2C), 129.98 (CH, C-1), 131.26 (CH, C-2), 136.74 and 141.25 (C, C1ʹ, 2C), 172.15 (C, *C*OCH_3_); EIMS *m*/*z* [M − H]^−^: 325 (100).

*5-Hydroxy-1,7-diphenylhept-1-ene-3-acetate* (**29**). Pale yellow gum, UV (MeOH) λ_max_ 251 nm; IR (KBr) *v*_max_ 3450 (OH), 1732 (C=O ester), 1238 (ester) cm^−1^; ^1^H-NMR (400 MHz, CDCl_3_): δ 1.60 (2H, m, H-6), 1.69 (2H, m, H-4), 1.97 (3H, s, acetate-Me), 2.69 (2H, m, H-7), 3.48 (1H, m, H-5), 5.57 (1H, m, H-3), 6.05 (1H, dd, *J* = 16.0, 6.8 Hz, H-2), 6.49 (1H, d, *J* = 16.0 Hz, H-1), 7.02–7.25 (10H, m, H-2ʹ–H-6ʹ); ^13^C-NMR (100 MHz): δ 21.13 (CH_3_, CO*C*H_3_), 32.09 (CH_2_, C-7), 38.80 (CH_2_, C-6), 43.08 (CH_2_, C-4), 66.72 (CH, C-5), 72.17 (CH, C-3), 125.85 and 128.08 (CH, C-4ʹ), 126.61 and 128.40 (CH, C2ʹ, 2 C, C4ʹ, 2C), 128.45 and 128.64 (CH, C-3ʹ, 2C, C5ʹ, 2C), 127.38 (CH, C-2), 132.17 (CH, C-1), 136.16 and 142.03 (C, C1ʹ), 171.52 (C, *C*OCH_3_); EIMS *m/z* [M − H]^−^: 325 (100).

#### 3.9.2. Isolation of Poilaneic Acid

Some 3.7 g of the crude MeOH propolis extract from Vilches was suspended in 120 ml CHCl_3_ and filtered to afford 3.2 g of CHCl_3_ solubles. The CHCl_3_ soluble mixture (3.2 g) was permeated on a Sephadex LH-20 column (column length: 52 cm; internal diameter: 2.5 cm). The column was eluted with dichlorometane (DCM)/Methanol (1:1 v/v) to afford 158 fractions of 12 mL each. After TLC comparison (silica gel, PE/EtOAc 6:4 as the mobile phase), fractions with similar TLC patterns were pooled into 14 groups. The main pools were fractions 30–35 (350 mg), 40–46 (845 mg), 50–59 (164 mg) and 70–77 (17 mg). The fraction pool 30–35 was further purified by silica gel chromatography (65 g silica gel, column length, 25 cm; internal diameter 2 cm). The column was eluted with a solvent gradient starting with PE/EtOAc (9:1 v/v; 500 mL), PE/EtOAc (85:15 v/v; 500 mL) and PE/EtOAc (8:2 v/v; 800 mL). Some 85 fractions were collected and pooled together according to the TLC pattern. The fraction pool 1–22 was purified by a preparative TLC (silica gel, DCM:toluene:diethyl ether 45:45:10 v/v/v) to afford poilaneic acid **34** (29 mg, Rf 0.45). The chloroform-soluble fraction pool 40–46 (500 mg) was chromatographed on silica gel (120 g; column length: 30 cm; internal diameter: 2.5 cm). The column was eluted with a solvent gradient starting with 900 ml of PE/EtOAc 7:3 v/v, then 500 mL PE/EtOAc 6:4 v/v, 200 ml PE/EtOAc 1:1 v/v and 200 ml EtOAc to afford after TLC comparison 30 fraction pools. The fractions 5–7 afforded pinocembrin **17** (107.7 mg), fractions 9–14 yielded chrysin **16** (98.3 mg) and fraction 27 contained a mixture of caffeic acid derivatives (32.0 mg). All the other fractions contained mixtures of compounds in low amounts and were not further investigated.

*Poilaneic acid* (**34**): pale yellow gum; ^1^H-NMR (400 MHz, CDCl_3_): δ 0.81 (3H, d, *J* = 6.8 Hz, H-16), 0.84 (3H, d, *J* = 6.8 Hz, H-17), 1.33 (1H, m, H-14a), 1.49 (1H, m, H-15), 1.65 (3H, s, H-19), 1.74 (1H, m, H-1), 1.78–1.80 (1H, m, H-14b), 1.81 (3H, s, H-18), 1.99 (1H, ddd, *J* = 13.2, 6.4, 3.6 Hz, H-13a), 2.02 (1H, ddd, *J* = 13.2, 6.4, 3.6 Hz, H-9a), 2.26 (1H, br d, *J* = 12.4 Hz, H-9b), 2.47-2.49 (1H, m, H-6a), 2.54 (1H, br d, *J* = 12.4, H-13b), 2.93 (2H, m, H-10), 3.09 (1H, m, H-6b), 5.17 (1H, d, *J* = 11.6 Hz, H-7), 5.21 (1H, dd, *J* = 15.6, 10.0 Hz, H-2), 5.57 (1H, br t, *J* = 7.2, H-5), 6.05 (1H, d, *J* = 10.8, H-11), 6.07 (1H, d, *J* = 15.6 Hz, H-3); ^13^C-NMR (100 MHz): δ 14.50 (CH_3_, C-19), 19.37 (CH_3_, C-16), 19.97 (CH_3_, C-18), 20.94 (CH_3_, C-17), 25.84 (CH_2_, C-10), 26.21 (CH_2_, C-6), 29.45 (CH_2_, C-14), 32.13 (CH_2_, C-13), 32.75 (CH, C-15), 38.52 (CH_2_, C-9), 47.91 (CH, C-1), 125.69 (CH, C-5), 127.74 (CH, C-7), 128.74 (C, C-12), 130.56 (CH, C-3), 131.01 (C, C-8), 131.32 (CH, C-2), 135.15 (C, C-4), 147.61 (CH, C-11), 172.83 (C, C-20); EIMS *m*/*z* [M − H]^−^: 301 (100), 257 (100).

### 3.10. Statistical Analysis

Determinations of TP, TF, DPPH and FRAP were performed two times in triplicate and results are expressed as mean values ± SD. For the TEAC assay, a curve was plotted for each sample and a correlation coefficient with 95% confidence limit was established. All statistical analyses were carried out using the software SPSS 14.0 for Windows.

## 4. Conclusions

The methanolic extract from the 19 propolis samples produced in the Región del Maule (central Chile) were compared for antibacterial activity, antioxidant effect and chemical composition. The samples showed common constituents but also some compounds that can be associated with specific ecosystems. While the present work offers a picture of the selected activities and chemical variability of propolis from central Chile, additional studies should be undertaken to disclose the potential of Chilean propolis for other uses. The results show the need of standardization of the propolis used to prepare commercial products to give support to the claimed benefits and uses. As the antimicrobial effect is not only associated with higher TP and TF content, fingerprint analysis of the constituents should be included and validated with appropriate assays. The chemical composition of the Chilean propolis shows clear differences with propolis from Bolivia, where phenolic-rich and triterpene-rich samples were recently reported and are used for the same commercial purposes [49].
